# Pan and Core Genome Analysis of 183 *Mycobacterium tuberculosis* Strains Revealed a High Inter-Species Diversity among the Human Adapted Strains

**DOI:** 10.3390/antibiotics10050500

**Published:** 2021-04-28

**Authors:** Fathiah Zakham, Tarja Sironen, Olli Vapalahti, Ravi Kant

**Affiliations:** 1 Department of Virology, Faculty of Medicine, University of Helsinki, 00014 Helsinki, Finland; fathiah.zakham@helsinki.fi (F.Z.); tarja.sironen@helsinki.fi (T.S.); olli.vapalahti@helsinki.fi (O.V.); 2 Department of Veterinary Biosciences, Faculty of Veterinary Medicine, University of Helsinki, 00014 Helsinki, Finland; 3 Faculty of Pharmacy, University of Helsinki, 00014 Helsinki, Finland; 4 HUSLAB, Hospital District of Helsinki and Uusimaa, 00260 Helsinki, Finland

**Keywords:** tuberculosis, *Mycobacterium tuberculosis*, BCG vaccine, comparative genome analysis, core genome, virulence, phylogeny

## Abstract

Tuberculosis (TB) is an airborne communicable disease with high morbidity and mortality rates, especially in developing countries. The causal agents of TB belong to the complex *Mycobacterium tuberculosis* (MTBc), which is composed of different human and animal TB associated species. Some animal associated species have zoonotic potential and add to the burden of TB management. The BCG (“*Bacillus Calmette-Guérin*”) vaccine is widely used for the prevention against TB, but its use is limited in immunocompromised patients and animals due to the adverse effects and disseminated life-threatening complications. In this study, we aimed to carry out a comparative genome analysis between the human adapted species including BCG vaccine strains to identify and pinpoint the conserved genes related to the virulence across all the species, which could add a new value for vaccine development. For this purpose, the sequences of 183 *Mycobacterium tuberculosis* (MTB) strains were retrieved from the freely available WGS dataset at NCBI. The species included: 168 sensu stricto MTB species with other human MTB complex associated strains: *M. tuberculosis* var. *africanum* (3), *M. tuberculosis* var. *bovis* (2 draft genomes) and 10 BCG species, which enabled the analysis of core genome which contains the conserved genes and some virulence factor determinants. Further, a phylogenetic tree was constructed including the genomes of human (183); animals MTB adapted strains (6) and the environmental *Mycobacterium* strain “*M. canettii*”. Our results showed that the core genome consists of 1166 conserved genes among these species, which represents a small portion of the pangenome (7036 genes). The remaining genes in the pangenome (5870) are accessory genes, adding a high inter-species diversity. Further, the core genome includes several virulence-associated genes and this could explain the rare infectiousness potential of some attenuated vaccine strains in some patients. This study reveals that low number of conserved genes in human adapted MTBc species and high inter-species diversity of the pan-genome could be considered for vaccine candidate development.

## 1. Introduction

Tuberculosis is an infectious airborne disease that (TB) evolved concomitantly with human 70,000 years and has killed millions of people in this time [1,2]. Currently, a quarter of the global population is infected with latent TB, and TB became the first leading infectious disease killer in the world [3]. The etiological agents of TB belong to the complex *Mycobacterium tuberculosis* (MTBc), which is composed of different human and animal TB associated species. The human-associated MTB includes eight lineages: MTB sensu stricto (L1–L4 and L7–L8) and *M. africanum* (L5 and L6) [4,5,6].

The animal-associated MTBc includes *M. bovis*, *M. microti*, *M. caprae*, *M. pinnipedii*, *M. orygis*, the dassie bacillus, *M. mungi*, *M. suricattae*, and the chimpanzee bacillus [5]. Some of MTB human associated species are very restricted geographically to certain regions of the world (L1, L5–L8), whereas others have a wider distribution and are considered as modern MTB species (L2–L4). The latter species are more virulent, causing severe manifestations and have many strains associated with anti-tuberculosis drug resistance [4].

According to the latest reports from the World Health Organization (WHO) in 2019, there were an estimated 10.0 million new TB cases and 1.2 million deaths worldwide [3]. Further, a zoonotic form of tuberculosis, caused by *M. bovis* (a member of MTB complex) added to the burden (an estimated 143,000 new cases with a range of 71,000–240,000 in 2018) and caused enormous economic losses [7]. *M. bovis* has a wide range spectrum of hosts, including domesticated animals like cattle, goats, pigs, cats, dogs, horses, and sheep [8], and also wild animals including brush tail possum, badger, buffalo, wood bison, wild boar, and white-tailed deer [9,10].

The MTB bacillus is highly infectious, contagious and has a long infectiousness period; a person with an active TB infection can infect 5 to 15 people via close or direct contact. Despite the international efforts to fight against TB, the problems of epidemic expansion and continuity of infection are still persistent. The reduced efficacy of the BCG vaccine and its limited use in animals and serious complications in immunocompromised patients [11]; altogether with the emergence of different forms of drug resistance lead to a massive number of deaths that exceeds that of HIV and malaria combined in the last 3 three years.

The uniqueness of the MTB bacilli is attributed to different phenotypic, immunologic, and genomic characteristics. Phenotypically, the MTBc species have acid fastness ability due to high lipid and mycolic acid content in the cell wall; they grow slowly on ordinary media cultures and have an obligate intracellular life style. Immunologically, the latency of the tubercle bacilli within the host is a remarkable feature due to their ability to evolve, construct new environmental niches and reshape the host cell signals to enhance stochastically their fitness and ability to reactivation and transmission [12].

Genomically, the MTBc members share high similarity at nucleotide level and 16SrRNA phylogeny analysis [8]. The MTB species are among the high GC content bacteria, which have different genes associated with the metabolism of lipids and cell wall, providing them the ability to escape macrophages. The members of the complex MTB, like other pathogenic mycobacteria, have been undergoing genome downsizing, losing many genes associated with free living style and keeping genes essential for pathogenicity, virulence and survival in the host cell [13,14]. Likewise, the MTBc members acquired new genes, adapting them to new host niche and overcoming its immune system barriers [13].

Several studies showed that MTB strains evolved from an ancestral-environmental mycobacterium, named *M. canettii* (smooth TB) through horizontal gene transfer, which is uncommon within MTBc species [4,13]. The regions of difference (RD) or large sequence polymorphisms (LSPs) have been considered as gold standards for the differentiation between the members of the MTBc and phylogenetic analysis [15]. Most of human adapted species have (RD1–RD10) regions, except *M. africanum* strains L6 sharing a common progenitor with animal-adapted species and lost the RD9 region. *M. africanum* (MAF) is restricted to the region of Western Africa; it was also isolated in African immigrants in industrialized countries. MAF has a closer relatedness to MTB sensu stricto strains in the pattern of RD than *M. bovis* [16]. Further, Ngabonziza et al. have recently found a sister clade (L8) of MTBc members, which is characterized by the lack of RD3, RD5, and RD14 [6]. L8 is restricted to the African Great lakes regions in Rwanda and Uganda and showed to be diverged prior to the loss of *cobF* gene, associated with vitamin B12 synthesis. The *cobF* gene is still available in *M. canettii* and some free living mycobacteria [6]. The animal adapted species also lack RD7, RD8, RD10 [4,17].

The human adapted modern MTB species (L2–L4) lost a specific region TbD1 including two genes *MmpS6* and *MmpL6*, which lead to virulence enhancement and global epidemic transmission through the resistance against oxidative stress and hypoxia [18].

In addition, all attenuated BCG vaccine strains lack the RD1(Rv3868 to Rv3875 and Rv3877), which encodes an ESX-1 secretion system [19]. This contains different genes related to virulence, mainly two important genes *esxA* and *esxB* encoding ESAT-6 and CFP-10 antigens, respectively [4,19]. By manipulating the parental BCG strains, new BCG vaccine strains were developed with different virulence levels and immunological efficacy [20].

Deepening in the genomics of human associated TB species and available vaccine strains could reveal new insights about the improvement of BCG efficacy and its use in human or animals. With the availability of ongoing whole genome sequencing datasets, comparative genomics tools could provide an insightful vision about the evolutionary events within an infectious agent and help to identify genes conserved across all the species and decipher the unique genes that give differential and special characteristics related to virulence and pathogenicity and consequently facilitate identifying new target genes for vaccine development [4,17].

In this study, we carried out a comparative genome analysis of 168 sensu stricto MTB species with other human MTBc: *M. tuberculosis* var. *africanum* (3), *M. tuberculosis* var. *bovis* (2), 10 BCG species through the freely available WGS dataset at NCBI, which enabled the analysis of core genome and virulence factors determinants across all the species. Then, a phylogenetic tree was constructed based on core genome including the genomes of human (183); some animals adapted MTB strains (6) and the environmental *Mycobacterium* “*M. canettii*”.

## 2. Materials and Methods

### 2.1. Genome Sequencing and Annotation

All the complete genomes of MTBc (181) and two draft genomes isolates (total of 183 genomes) were obtained from NCBI: http://www.ncbi.nlm.nih.gov/ (accessed on 2 February 2020) GenBank, available sequences on 2 February 2020 were retrieved and analyzed for the purpose of comparative genome analysis. The species includes 168 sensu stricto MTB species with other human associated TB strains: *M. tuberculosis* var. *africanum* GM041182, *M. tuberculosis* var. *africanum* UT307, *M. tuberculosis* var. *africanum* strain 25, *M. tuberculosis* var. *bovis* MBE9, and *M. tuberculosis* var. bovis strain MAL010093 (draft genome were included) *M. tuberculosis* var. *bovis* BCG strains (10). For the phylogeny analysis, the genomes of some animal adapted-MTB strains (*M. tuberculosis* var. *bovis* strain AF2122/97, *M. mungi*, *M. orygis*, *M. tuberculosis* var. *microti*, *M. tuberculosis* var. *caprae* and *M. tuberculosis* var. *pinnipedii*) and the environmental *Mycobacterium* strain “*M. canettii*” were included. *M. tuberculosis* var. *bovis* strain AF2122/97 and “*M. canettii*” have complete genomes and the remaining animal strains have draft genomes.

### 2.2. Orthologous Gene Prediction and Genome Sequence Comparison

Orthologous proteins for 184 *Mycobacterium tuberculosis* genomes were identified by the comparison of all the species against each other by the use of blastp and the application of the default scoring matrix BLOSUM62 and an initial-value cut-off of 1 × 10^−5^. Normalizing of the raw BLAST hit scores against the maximum possible score (defined here as the self-hit score for each gene) was performed. This resulted in a score ratio value (SRV) between 0 and 100 that showed the quality of the hit much better than the raw blast bit score.

Two proteins were considered orthologous if a reciprocal best blast hit existed between them, and both hits had an SRV > 32. The SRV threshold is computed from distribution of blast hits between analyzed sequences as described in the supplement of Blom et al. (2009) [21]. Based on this orthology principle, the core genome was calculated as the set of genes that had orthologous proteins in all other analyzed strains.

The pan-genome was estimated as the set of all unique proteins of a set of genomes. All proteins of one reference genome were considered the basic set for the calculation. Afterwards, the proteins of a second genome were matched with this set, and all proteins in the second genome that had no orthologous proteins in the starting proteins set were added to this set. This process was iteratively repeated for all genomes of the compared set, leading to the pan-genome.

### 2.3. Phylogenetic Tree Construction

A phylogenetic tree was constructed with a modified version of the pipeline designed by Zbodnov and Bork as described by blom et al. (2009) [21]. Alignments of the core gene sets were compiled using MUSCLE [22], the numerous resulting multiple alignments were concatenated, and poorly aligned positions were removed using GBLOCKS [23]. A trimmed multiple alignment system was used to create a phylogenetic tree using the neighbor-joining operation of PHYLIP [24].

## 3. Results

### 3.1. General Features

In this study, we present a comparative genome analysis of 183 MTB strains obtained from complete genome (181) and draft genome sequences (2) of human MTB adapted strains. Additional sex genomes of different animal MTB-adapted strains and *M. canettii* were also considered for the phylogeny analysis.

The length of the complete genomes ranged between 4.3 to 4.4 MB, with a number of genes between 3670 to 4826 with a high GC content (65.01–65.07%). The numbers of predicted protein-encoding open reading-frames proteins ranged between 3622 to 4778 (Supplementary Table S1 summarizes a number of characteristics for each genome).

### 3.2. Core and Pan Genome

The pan genome compares the genomes of different strains from the MTB complex as the mean for determining the overall genetic content of a given species. Several genetic loci from the pan genome needed for the bacterial survival forms the core genome of a particular species. Most of these genes are essential for basic housekeeping functions. The dispensable genome of a species consists of the genetic content of a species, which is present only in a subset of strains and is believed to play important roles in phenotypic variation and genome evolution. The pan genome of 183 complete and draft genomes of MTB strains consists of 7036 genes (Table S2) of which 1166 (16.6%) formed the core genome (Table S3). Out of the 1166 core genes 347 were either hypothetical proteins or proteins with unknown function. The remaining genes are essential genes associated with replication, DNA repair, protein translation and regulation, synthesis of mycolic and fatty acids, transcriptional regulation, energy metabolism, catabolism, virulence, and other functions.

**Figure 1:**
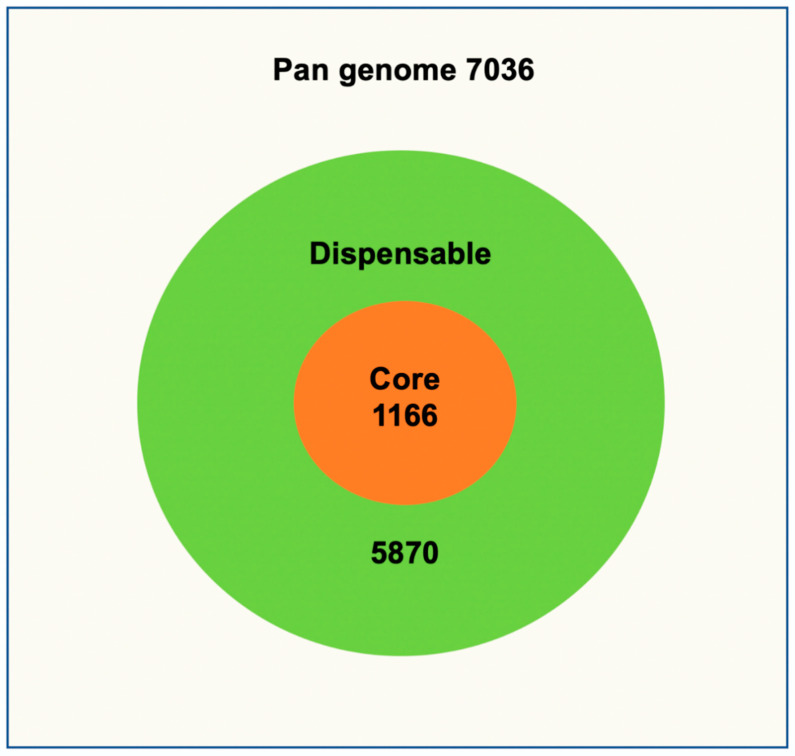
MTB pan/core genome. The core genome is listed with orange background and the all the remaining genes (dispensable) part of the pan genome are listed with the green background.

The dispensable genome of entire MTB strains consists of 5870 genes revealing high inter-species diversity (Figure 1). Furthermore, a careful study of the pangenome development data revealed an α-value of 0.872, which is rather high, indicating that the pangenome curve starts to flatten at approximately 120 genomes. Genomes added after that contribute only few genes to the pangenome implying the pangenome of 183 MTB strains is eventually proceeding to a closed status representing the entire genetic repertoire of MTB species. A similar pattern was observed with the core genome development plot.

### 3.3. Phylogenetic Analysis

**Figure 2:**
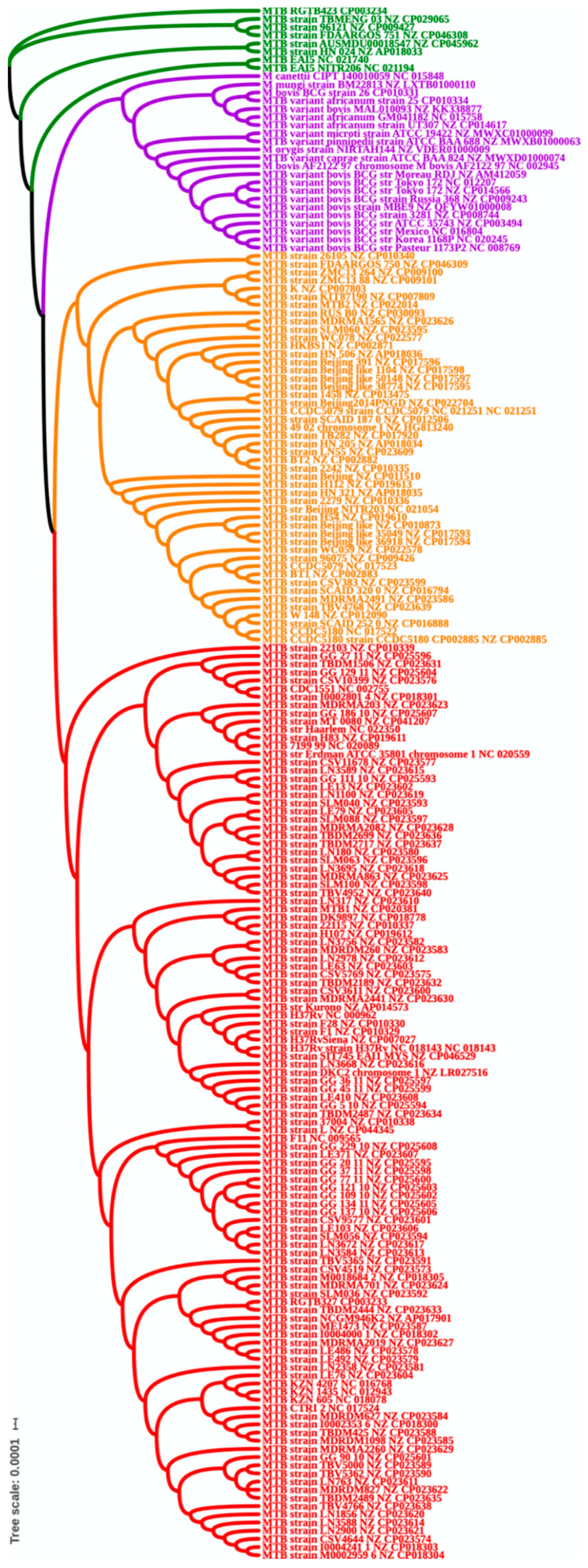
Phylogenetic tree based on core genome.

A phylogenetic tree was constructed based on the extracted multiple alignment of the 1059 core proteins from all 190 genomes. The results of phylogenetic analyses are shown in Figure 2; remarkably, based on the core genome phylogenetic tree, the 190 (human and animal MTB adapted strain) MTBc strains were grouped into four clades (Figure 2). The first, third and fourth clades were composed of sensu stricto MTB species. However, *M. tuberculosis* var. *africanum*, *M. tuberculosis* var. *bovis* human adapted strains were clustering with animal adapted strains and *M. canettii*”, showing a shared common ancestor.

The vertical line at the beginning of Figure 2 (in this case, 0.01) is used to provide a rough measure of genetic distance. Furthermore, the pattern of clustering confirms a high similarity between the MTBc members and is in accordance with other previous studies. No clear clustering emerged from the geographical location or niche of these strains.

### 3.4. Clade Specific Analysis Based on Phylogenetic Tree

Four clades derived from the phylogenetic tree and represented in different colors in Figure 2. These clades were inspected for clade specific genes to investigate if there is something specific in these clades that lead to four different clusters in the phylogenetic tree. For each clade we looked for genes present in all the genomes of a particular clade but absent in all other MTB strains. Apart from a few hypothetical proteins, the clade specific analysis did not produce any significant set of genes specific to each clade. The analysis of each clade species shows very high similarity among MTB strains. Further, we checked 14 Regions of RD and also TbD1, which are all absent in the core genome.

However, some of the remaining genes are related to pathogenicity and virulence and Table 1 showed all the virulence related genes in this collection.

## 4. Discussion

Tuberculosis is the leading cause of death as an infectious agent and *M. tuberculosis*, the etiological agent succeeded to co-evolve, adapt, and interact reciprocally with the human beings through the years. The human adapted MTBc strains share a high relatedness at genomic level and differ in geographic distribution, virulence, transmissibility, and drug resistance pattern. Currently, the MTBc species: *M. tuberculosis* var. *africanum*, *M. tuberculosis* var. *bovis*, *M. tuberculosis* var. *caprae*, *M. tuberculosis* var. *microti*, and *M. tuberculosis* var. *pinnipedii* are considered as heterotypic variants of *M. tuberculosis* [26]. In addition, *M. canettii M. orygis* and *M. mungi* are strains of the species *M. tuberculosis* [26]. There have been several pan/core genomic studies on MTBs but most have been based on fewer numbers of genomes [27,28]. Other large-scale studies on core/pan-genome analysis showed the utility of this tool to study the genetic difference within species and to identify the known and novel genetic signature of genes conferring resistance [29,30].

Thus, we decided to take all the complete genomes of MTBs available and report the complete genetic repertoire of the species. In the present study, we carried out a comparative genome analysis of 183 MTBc human associated species including 168 sensu stricto MTB species, *M. tuberculosis* var. *africanum* (3), *M. tuberculosis* var. *bovis* (2), 10 *M. tuberculosis* var. *bovis BCG* vaccine species through the freely available WGS dataset at NCBI, which enabled the calculation of the core-genome across all the species and to reveal the main virulence conserved genes. Then, based on the core genome, we constructed a phylogenetic tree by the use of human (183), animals MTB adapted strains (6), and the environmental *Mycobacterium* pathogen “*M. canettii*”.

Genome size, number of genes, and number of proteins varied according to the species, and thus considerably correlated with size of pan genome consisting of 7036 genes, which comprised all the genes encoding proteins in all the species including the accessory genes and core genome. In contrast, the core genome of 1166 genes was represented by the minimal set of indispensable conserved genes across all the species [14]. Even though, the number of core genes are only about 17% of the overall pan genome this is still quite large and in line with previous studies. These core genome genes provide the identity of MTBc human associated species and exist in each strain included in this study. As expected, all the 14 Regions of RD and TbD1 that have been used for the discrimination between the MTBc lineages are absent in the core genome, which includes only conserved genes across all the species. Most of the conserved genes are associated with DNA replication, repair, translation, transcription regulation, synthesis of fatty acids and mycolic acid, and metabolism and catabolism of macromolecules. In a previous study, Yang et al. studies 49 MTB, *M. bovis* and BCG vaccine species and identified 3679 conserved genes and only 1122 accessory genes, which contradict our study and reveal the importance of including more strains for having a clear vision about the evolution of MTBc strains. Some of the genes in the core-genome were among the optimal required genes for the in vitro and in vivo growth stated by Zhang et al., 2012 and Sassetti et al., 2003 [31,32]. However, all those genes were available in the pan-genome. Both studies were based on the analysis of the virulent reference strain *M. tuberculosis* H37 Rv.

The core genome results of human adapted MTBc strains showed that BCG vaccine strains still share several genes associated to virulence and persistence of pathogen within the host.

For instance, despite the absence of RD1 genes encoding the Esx1 of the secretion system in the core genome, the *espC* gene is still conserved in all the MTBc species included in this study. The *espC* has a high similarity in sequence, size and an equivalent immunodominancy to CFP-10 and ESAT-6, which are able to engender cellular immunity [33]. The Phthiocerol dimycocerosates (PDIM) is another putative virulence factor in the MTBc complex members and other slow growing mycobacteria and plays an essential role in the phagosomal rupture induced by the tubercle bacilli [4]. The conserved *drrABC* operon (daunorubicin ABC transporter) and especially the drrC, which is involved in the transport of PDIM indicates a certain level of virulence among the species. Significantly, mutant MTB strains harboring inactivated *drrB* and *drrC* were not able to secrete PDIM [34].

The PhoR or the two-component system response sensor kinase is also among the conserved virulence genes found in the core genome. In addition to its role as a transcriptional regulator, this gene showed a key evolutionary role in MTBc host speciation after a functional divergence followed by positive selection from the common ancestor and transition from free living to a host specific intracellular parasitic life style [35]. Interestingly, the DU2-III BCG vaccine strains like Glaxo and Danish (not included in this study) showed a deletion of 10 nucleotides in codon 91 of PhoR, which could be associated with more attenuation in these strains [36].

BCG vaccine has been used for several years for the prevention of different mycobacterial diseases like TB, Bruli ulcer, and leprosy. It is also useful for the treatment of some non-communicable diseases, mainly an adjunctive biotherapy bladder cancer. Most recently, it showed to have some efficacy against COVID 19 [37]. Despite the deletion of the RD1 region that lead to the attenuation of its infectivity, the BCG vaccine could be disseminated in some cases and provoke life-threatening infections in immunocompromised patients including HIV-infected and severe combined immunodeficiency (SCID) patients. In 2007, the WHO stopped recommending the use of BCG vaccination for HIV infected children due to the deleterious risk of BCG disseminated infection or BCGosis, which could be engendered by its use [11]. Recently, it showed also the same effect on cancerous patients treated with or without intravesical instillation, causing pulmonary TB, which is sometimes associated with extrapulmonary manifestations and very serious complications [38,39].

The development of new vaccines is challenging in the field of mycobacteriology and ensuring the safety and efficacy and lowering the adverse effects of a vaccine are major health priorities with the emergence of multi drug resistant strains and incurable forms of TB. Recent comparative genome analysis of BCG vaccine strains showed that engineering the BCG strains through the years lead to the loss of important component including T-cell epitopes and restoring and manipulating some epitopes could offer new solutions for vaccine development [20]. Reintroducing some deleted RD regions or some of their proteins in new BCG vaccine candidate could stimulate and increase the protective immunity [40]. For instance, a recent study showed that the integration of the RD4 (Rv1506c-Rv1516c) in BCG vaccine strain increased the protection against *M. marinum* in zebrafish model [41].

Our phylogenetic analysis is in accordance with other several studies; showing a high similarity and clustering pattern between the MTBc members and confirming that the MTBC members are sharing the same ancestor in their evolutionary events and a high relatedness [13]. The MTBc strains were grouped into four clades. The first, third and fourth clades included MTB sensu stricto and the second clade is represented by *M. tuberculosis* var. *africanum* (L5 and L6), *M. tuberculosis* var. *bovis*, all the animal adapted MTBc strains and all the BCG vaccine strains. BCG vaccine strains are attenuated derivatives *M. tuberculosis* var. *bovis* and share very high similarity with each other despite their immunogenicity and virulence level.

Most modern MTBc species from lineages L2, L3, and L4 are world-widely distributed, more virulent and many of them associated with drug resistance, especially Beijing strain (L2). The results of the clade specific analysis based on phylogenetic tree are also with concordance with the phylogeny results and confirm that all the species have a distinct and common pathway of the evolutionary bottleneck and a clonal expansion toward more speciation to the intracellular niche of human macrophages [42].

The core genome represents only 16.6% of the pan genome, which indicates that most acquired genes are conferring more functionality, complexity, and diversity. However, our results showed that the pan genome was flattened after 120 genomes and proceeded to a closed pan genome status, confirming an allopatric and sympatric geographic speciation of MTBc complex members, which correlates with pathogen–host interactions and this explains the different severity and persistence patterns of MTB species within the host [43].

## 5. Conclusions

To conclude, our results showed that despite the high phylogenetic relatedness between the MTBc species, the core genome represents only a small portion of the pan-genome and still contains several virulence factors, which can be exploited for further diagnostic and vaccine development.

## Figures and Tables

**Table 1:** Virulence conserved genes in the core genome, according to Forrellad et al. [25].

RV Number or Gene in the Core Genome	Role of Virulence Factor
Rv3615c (*espC*), Rv3867 (*espH*), Rv1539 (*lspA*), Rv1791	Secretion system
Rv2936 (*DrrA*), Rv2937 (*DrrB*), Rv2938 (*drrC*): daunorubicin ABC transporter	Synthesis of Phthiocerol dimycocerosates (PDIM)
Rv0642c (*mmaA4*)	Mycolic acid synthesis (hydroxymycolate synthase)
Rv3568c (*hsaC*), Rv3541c	Catabolism of cholesterol
Rv0167, Rv0170, Rv0173 (mce operon 1), Rv0589 (part of operon Mce2A), Rv0199	Cell wall and conserved membrane proteins
Rv1410c, Rv1235 (*lpqY*)	Lipoproteins
Rv2031c, *acr1* alpha-crystallin *(hspX)*	Inhibition of macrophage effectors
Rv1941, Rv1932 (*tpX*), Rv2234 (*ptpA*)	Oxidative/nitrosative stresses
Rv3133c (*devR*), Rv3132c (*dosR*), Rv2745c (*clgR*), Rv0353 (*hspR*), Rv3416 (*whiB3*), Rv0348 (*mosR),* Rv3082c (*virS*), Rv0821c *PhoY2*, Rv0990c, Rv0491 (*regX3*)	transcriptional regulators
Rv2115c (*mpa*)	Mycobacterial proteasome ATPase
Rv0758 (*phoR*), sensor kinase of phosphate regulon	Gene Expression Regulator
Rv2069 (*sigC*), Rv3414c (*sigD*), Rv1221 (*sigE*), Rv3223c (*sigH*), Rv0735 (*sigL*)	Sigma factors: RNA polymerase sigma factors
Rv2711 (*ideR*) iron-dependent repressor and activator Rv1811 (*mgtC*) Mg2+ transport P-type ATPase	Metal importers
Rv0990c conserved heat shock protein (*hsp22.5*)	Other mycobacterial virulence factors

## Data Availability

The datasets generated for this study can be found in the main manuscript and supplementary files.

## Supplementary Materials

The following are available online at https://www.mdpi.com/article/10.3390/antibiotics10050500/s1, Table S1. A general overview of 190 MTB genomes. Table S2. The pan genome of 183 MTB genomes. Table S3. The core genome of 183 MTB genomes.
